# Azithromycin for acute bronchiolitis and wheezing episodes in children – a systematic review with meta-analysis

**DOI:** 10.1038/s41390-023-02953-z

**Published:** 2023-12-08

**Authors:** Rosa-Maria Ukkonen, Marjo Renko, Ilari Kuitunen

**Affiliations:** 1 Institute of Clinical Medicine and Department of Pediatrics, University of Eastern Finland, Kuopio, Finland; 2 Department of Pediatrics, Kuopio University Hospital, Kuopio, Finland

## Abstract

**Background:**

The aim of this systematic review and meta-analysis was to analyse the efficacy of azithromycin in acute bronchiolitis and wheezing.

**Methods:**

PubMed, Scopus, and Web of Science databases were searched for randomized controlled trials comparing azithromycin to placebo in children <2 years of age. Main outcomes were progress of acute wheezing episode and recurrence of wheezing. We used random-effects model to calculate mean difference (MD) with 95% confidence interval (CI) or risk ratios (RR) with CI.

**Results:**

We screened 1604 abstracts and included 7 studies. Risk of bias was low in three and had some concerns in four studies. Need for intensive care unit treatment was assessed in four studies (446 children) and the risk difference was 0.0% (CI –2.0 to 2.0; low quality evidence). Hospitalization duration was –0.27 days shorter in the azithromycin group (MD-0.27, CI –0.47 to –0.07; three studies; moderate quality evidence). Azithromycin did not prevent recurrence of wheezing (RR 0.84, CI 0.45–1.56; three studies), hospital readmissions (RR 1.14, CI 0.82–1.60; four studies).

**Conclusions:**

We found moderate quality evidence that azithromycin may reduce hospitalization duration. Low certainty evidence suggests that azithromycin does not reduce the need for intensive care unit treatment. Furthermore, azithromycin did not prevent wheezing recurrence.

**Impact:**

- Azithromycin may reduce hospitalization time in acute bronchiolitis and wheezing episodes among children aged less than two.
- Azithromycin administrated during the acute wheezing period, does not have preventive effect on wheezing recurrence.
- Azithromycin seemed to have similar adverse event profile than placebo.
- Future studies with clinically relevant outcomes, and sufficient sample sizes are needed, before implementing azithromycin into clinical use.

## Introduction

One-third of children suffer from wheezing during the first three years of life.^1^ Up to 26% of children present with recurrent wheeze (≥ 3 episodes) by age 6.^2^ Acute bronchiolitis causes the majority of hospital admissions in infants under 12 months in the United States and over three million hospital admissions annually worldwide, and predisposes to subsequent recurrent wheezing and asthma development.^3–5^ Bronchiolitis in infants under 12 months is mainly caused by respiratory syncytial virus (RSV), whereas rhinovirus (RV) prevails in older children.^6,7^

Because of the strong association to viral respiratory infections, guidelines do not suggest antibiotics for wheezing, yet they are widely used.^8^ Only supportive treatment of breathing and oxygenation, fluid replacement and alleviating symptoms, usually with inhaled short acting beta-agonists for children older than 12 months are widely accepted. Previously explored pharmacological prevention of post-RSV wheezing include montelukast and corticosteroids both inhaled and systemic. The results have remained mostly negative, possibly owing to the fact that the inflammation pattern in bronchiolitis and wheezy bronchitis is predominantly non-eosinophilic. Inhaled beta-agonists do not reduce hospital admissions or length-of-stay.^9,10^ Novel treatment strategies are needed to attenuate and prevent the symptoms of wheezing.

The exact shares of viral and bacterial aetiologies of wheezing remain obscure, as viral isolation is costly and not part of the routine examination.^11^ In the COPSAC_2000_ birth cohort the prevalence of co-infection of virus and bacteria was 55% in acute wheezing episodes among young children (85% either *Haemophilus influenzae*, *Streptococcus pneumoniae* or *Moraxella catarrhalis*), suggesting that antibiotics might have a role in treating such episodes to some extent.^12^ In adults macrolides have been successfully used in the treatment of chronic pulmonary diseases, such as diffuse panbronchiolitis, asthma, bronchiectasis, cystic fibrosis, and acute exacerbations of chronic obstructive pulmonary disease.^13^ A recent study respectively found that azithromycin treatment in children with poorly controlled asthma resulted in reduced asthma symptoms and exacerbations.^14^ Pulmonary diseases with neutrophil dominance are the most responsive to macrolide treatment. Macrolides have been long used for their antibacterial activity that extends over *Mycoplasma pneumoniae* and *chlamydia pneumoniae*, and their additional antiviral and anti-inflammatory effects have been studied during the past two decades.^15–19^ The anti-inflammatory properties are linked to the modulation of interleukin and TNF-α release and thus to the inhibition of the chemotaxis, oxidative burst and endothelial adhesion of neutrophiles. Immunomodulatory effects on several other cells, including fibroblasts, epithelial and endothelial cells, macrophages, and dendritic cells, have also been reported. Azithromycin has the highest tissue penetration of macrolides, accumulating particularly in phagocytes, which carry azithromycin to the inflammation site.^19^ Azithromycin also increases epithelial integrity.^20^

In addition to these well-targeted effects on the previously mentioned adult inflammatory respiratory diseases, azithromycin is a well-tolerated and safe drug, and therefore has attracted interest in its suitability for treating wheezing in children. In the past two decades a handful of studies have investigated this issue, but the results remain controversial. A couple of studies have demonstrated promising protection against subsequent wheezing episodes in infants treated with azithromycin during the initial episode.^9,21^ Stokholm et al. found out that azithromycin caused an impressive shortening of 63% in the asthma-like symptom episode.^22^ Bacharier et al. reported that the risk of progression to severe LRTI was 36% lower among azithromycin group compared to placebo.^18^

In this meta-analysis we assess the clinical effect of azithromycin in treating acute viral bronchiolitis or other wheezing episode (all later referred as wheeze or wheezing) and preventing recurrent episodes in children ages less than 24 months.^10,23,24^

## Methods

### Search strategy

A comprehensive search was conducted in December 2022 to following databases: PubMed (MEDLINE), Scopus, and Web of Science. The complete search strategy is presented in the supplementary file 1. We did not use any filters in the search in the PubMed and Web of Science databases. In Scopus we used language filter and filtered only to articles. There were no limitations regarding the time of publication.

### Inclusion and exclusion criteria

We included randomized controlled trials (RCTs) comparing azithromycin treatment regardless of dose, route of administration and course duration to placebo or no intervention added to standard care in children aged less than 24 months suffering from acute wheezing. Acute wheezing was defined as clinically or parentally diagnosed obstructive expiratory respiratory disorder and it included diagnoses of bronchiolitis, and recurrent wheezing. We included studies with children less than two years of age, as the prevalence and incidence of wheezing is highest in this age-group. Study outcomes had to be clinical as studies only focused on laboratory parameters were excluded. Furthermore, all studies that did not report original data or were observational were excluded. Non-English reports were also excluded.

### Review process

Covidence software was used in the screening and extracting process. Every abstract and full text was screened by two individual authors (RMU and IK) Disagreement between the two screening authors was resolved by a third author opinion or mutual consensus. Two authors (RMU and IK) performed data extraction independently. Following information was extracted: authors, year of publication, country where the study was conducted, study period, study design, original inclusion criteria, the definition of intervention group and control group, total number of patients included in the study, number of patients in the intervention and control groups, and outcome measures.

### Main outcome

Our main outcomes were: (1) how azithromycin impacted the progress of acute wheezing episode, meaning need for hospitalization, need for pediatric intensive care unit treatment and length of hospital stay. (2) Did azithromycin have an impact on the recurrence of wheezing, including novel wheezing episodes, need for readmission to hospital and asthma diagnoses during the follow-up. Our secondary outcome was the treatment related adverse events. The main effect measures were risk ratios (RR) with 95% confidence interval (CI) for dichotomous outcomes and mean difference (MD) with CI for continuous outcomes. Risk difference (RD) with CI was used for outcomes with extremely low event rates.

### Risk of bias

Cochrane risk of bias tool 2.0 was used to evaluate the quality of included studies, and risk of bias figures were reported accordingly. Risk of bias was performed by one author (IK). Robvis package in R version 4.2.2 was used to produce the figures.

### Statistical analysis

The RevMan version 5.4.1 were used for the meta-analysis. Data analysis was performed according to Cochrane Handbook of Systematic Reviews Guidelines. Forest plots were presented for all pooled outcomes. We chose a random-effects model for main analyses due to assumed high heterogeneity based on the inclusion criteria and interventions (azithromycin dose, route of administration, course length and study setting). Statistical heterogeneity was tested and I^2^ results are reported in the forest plots. The model decision between fixed-effects and random-effects was however unrelated to statistical heterogeneity (I^2^ value). We used Mantel-Haenszel method in analyses. Furthermore, in one study we pooled high dose and standard dose azithromycin groups together for analysis in terms of reported mean and SD and Cochrane formula was used for this calculation.

We have reported our findings according to Preferred Reporting Items in Systematic Reviews and Meta-Analysis (PRISMA). PRISMA checklist is found in the supplementary materials. The body of evidence for each of the main outcomes were assessed according to GRADE (Grading of Recommendations, Assessment, Development and Evaluations) framework.

#### Protocol registration

We registered our protocol in Prospero (registration no. CRD42023392184).

## Results

### Search results

**Fig. 1:**
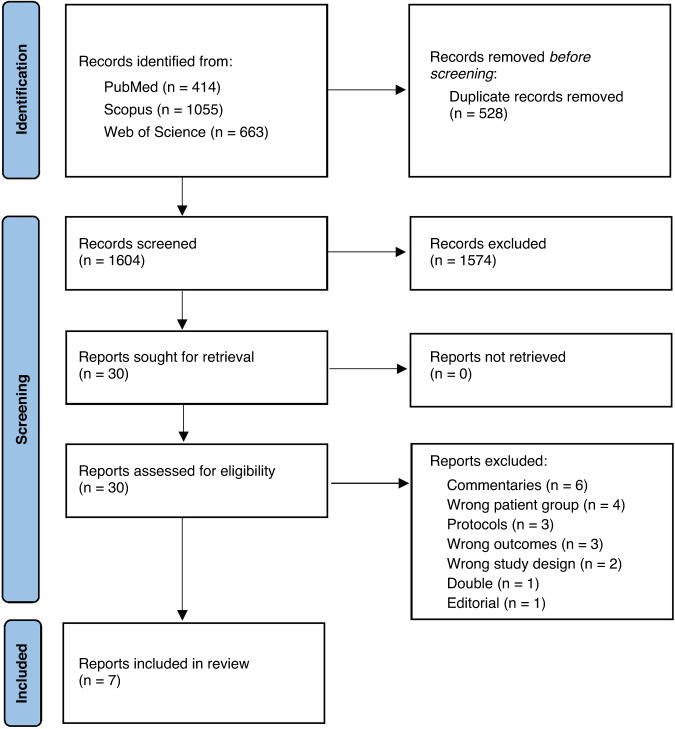
PRISMA flowchart of the study selection process.

After screening 1604 abstracts and further assessment of 30 full reports, we included 7 studies for systematic review and meta-analysis (Fig. 1).

### Characteristics of the included studies and patients

All included studies were double blinded RCTs and they were performed in the USA, Brazil, Australia, and Netherlands. (Table 1) The azithromycin dosing was mostly 10 mg/kg daily, and the duration varied from 3 days to 14 days. Two studies used a strategy with single or weekly repeated higher doses (30 mg/kg). (Table 1) Most of the patients were otherwise healthy children diagnosed with an acute bronchiolitis, mild or severe. (Table 1) Three studies focused on RSV cases and four included all viral aetiologies (Supplementary Table 1 and Table 1). The inclusion and exclusion criteria in the included studies were rather similar (Supplementary Table 1).

**Table 1:** Background characteristics of the included studies.

Study	Country	Design	Blinding	Dosing	Microbiology diagnostics	Participants (N)		Age	Main outcome
						Intervention	control		
Kneyber et al. 2007	Netherlands	RCT	Double, placebo	Daily dose of 10 mg/kg for 3 days	All RSV positive	32	39	≤24 months	Length of stay
Pinto et al. 2012	Brazil	RCT	Double, placebo	Daily dose of 10 mg/kg for 7 days	RSV diagnostics performed	88	96	<12 months	Length of stay Duration of supplemental O2 requirement
McCallum et al. ^33^	Australia	RCT	Double, placebo	Single dose of 30 mg/kg	Multiplex panel PCR viral testing	50	47	≤18 months	Length of stay Duration of supplemental O2 requirement
Beigelman et al. ^9^	USA	RCT	Double, placebo	Daily dose of 10 mg/kg for 7 days, followed by daily dose of 5 mg/kg for another 7 days	All RSV positive	20	20	1–18 months	Serum and nasal lavage fluid IL-8 levels 2 or more additional wheezing episodes
McCallum et al. ^10^	Australia	RCT	Double, placebo	Three weekly doses of 30 mg/kg	Multiplex panel PCR viral testing + nasopharyngeal swab bacterial culture	106	113	≤24 months	Length of stay Duration of supplemental O2 requirement
Luisi et al. ^21^	Brazil	RCT	Double, placebo	Daily dose of 10 mg/kg for 7 days	Multiplex panel PCR viral testing	50	54	<12 months	Recurrent wheezing and hospital readmissions
Beigelman et al. ^30^	USA	RCT	Double, placebo	Daily dose of 10 mg/kg for 7 days, followed by daily dose of 5 mg/kg for another 7 days	All RSV positive	101	99	1–18 months	Occurrence of a third episode of parent-reported wheezing after the index episode

### Risk of bias

**Fig. 2:**
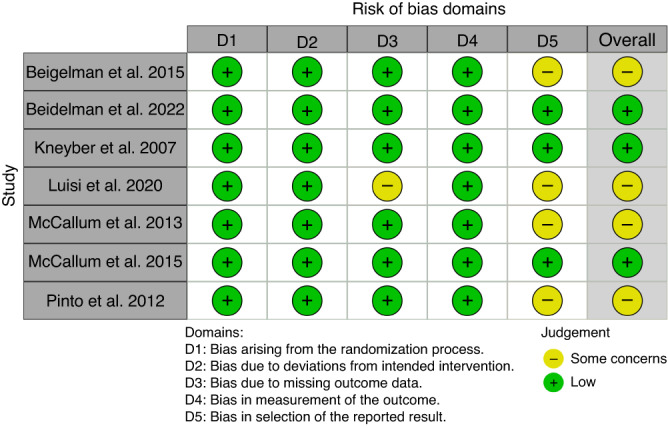
Risk of bias in the individual studies assessed in five domains and overall.

Risk of bias was assessed to be low in three studies and had some concerns in four studies (Fig. 2) Most of the bias was due to selection of reported results. One study had some concerns with missing outcome data. However, none of these were judged to create such issues to validity that the study would have assessed to be in high risk of bias.

### Clinical course of acute wheezing episodes

**Fig. 3:**
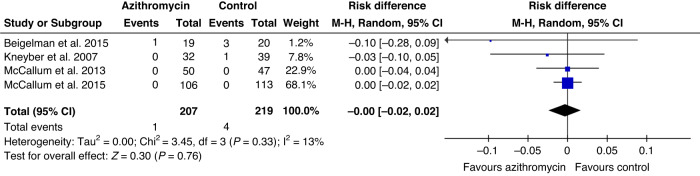
Forest plot of the need for pediatric intensive care unit admission. Need for pediatric intensive care unit admission.

**Fig. 4:**
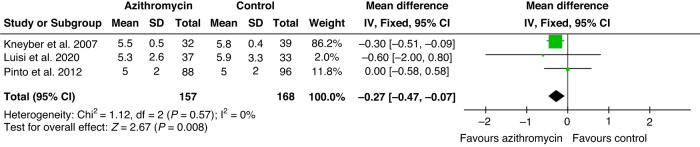
Forest plot of the hospitalization length. Hospitalization length in days.

Four studies (446 children) analyzed the need for PICU admission and one child in the azithromycin group (0.5%) and four children in the control group (1.8%) were admitted to PICU (pooled RD 0.0%, CI –2.0% to 2.0%; Fig. 3). Evidence quality for PICU admissions was ranked as low. Three studies with 325 children analyzed the overall length of hospitalization period, and the mean difference was -0.27 days (CI –0.47 to –0.07 days; Fig. 4) favoring azithromycin group. Evidence quality for hospitalization duration was ranked as moderate (Table 2).

**Table 2:** Summary of findings table and GRADE assessment.

Outcome	N of patients (N of studies)	Absolute effect		Relative effect	GRADE
		Azithromycin	Control		
Progress of acute wheezing episode					
Need for PICU admission	446 (4)	1 of 207 (0.5%)	4 of 219 (1.8%)	RD 0.0% (CI –2.0% to 2.0%)	Low*
Hospitalization duration	325 (3)	Not applicable	Not applicable	MD -0.27 days (CI –0.47 to 0.07)	Moderate**
Wheezing recurrence					
Novel wheezing episodes	297 (3)	59 of 152 (38.8%)	56 of 145 (38.6%)	RR 0.84 (CI 0.45–1.56)	Very Low***
Hospital readmissions	585 (4)	58 of 296 (19.6%)	51 of 289 (17.6%)	RR 1.14 (CI 0.82–1.60)	Low*
Asthma diagnosed	227 (2)	16 of 115 (13.9%)	14 of 113 (12.4%)	RR 0.94 (CI 0.29–3.10)	Low*
Adverse events					
Treatment related adverse events	515 (3)	16 of 257 (6.2%)	17 of 258 (6.6%)	RD 0.0% (CI –2.0% to 2.0%)	Low*
Gastrointestinal adverse events	268 (2)	9 of 135 (6.7%)	8 of 133 (6.0%)	RD 2.0% (CI –1.0% to 5.0%)	Low*

^*^Downgraded due to imprecision and risk of bias. ** Downgraded due to risk of bias. *** Downgraded due to imprecision, inconsistency, and risk of bias.

*CI* confidence intervals, *MD* mean difference, *RD* risk difference, *RR* risk ratio.

### Prevention of recurrent wheezing

Two studies analyzed the readmission rate at three months follow-up and 13.8% (12/87) were readmitted in the azithromycin group and 13.8% in the control group (11/80), RR 1.01 (CI 0.48–2.15; Supplementary Fig. 1). Three studies assessed the need for readmission at six months and rates were 23.2% in the azithromycin group and 20.6% in the control group, RR 1.16 (CI 0.79–1.69; Supplementary Fig. 1). One study further assessed the readmission during one-year follow-up and the corresponding rates were 10.5% and 5.0% (RR 2.11, CI 0.21–21.36; Supplementary Fig. 1). When all of the previous timepoints were pooled together the risk ratio for readmission to hospital was 1.14 (CI 0.82–1.60; Supplementary Fig. 1). Evidence quality was ranked as low (Table 2).

Wheezing recurrence without the need for inpatient admission was analyzed in three studies and in three different time points. One study assessed recurrence at three months and the RR for wheezing was 0.48 (CI 0.22–1.06); Supplementary Fig. 2. One study assessed the recurrence during one year follow-up (RR 0.74, CI 0.35–1.54; Supplementary Fig. 2). One study analyzed recurrence up to 4 years of follow-up and the RR was 1.31 (CI 0.92-1.85). After pooling of these studies, the risk ratio for at least one recurrence of wheezing episode was 0.84 (CI 0.45–1.56; Supplementary Fig. 2). Evidence quality was ranked as very low (Table 2).

Two studies assessed the clinically diagnosed asthma. At one year, the RR was 0.42 (CI 0.09–1.92, one study) and at up to four years 1.49 (CI 0.68–3.28, one study). Pooled estimate for asthma did not show any difference between the groups (0.94, CI 0.29–3.10; Supplementary Fig. 3). Evidence quality was ranked as low.

### Adverse events

Three studies with 515 children analyzed treatment related serious adverse events and reported 16 (6.2%) events among the 257 children in the azithromycin group and 17 (6.6%) events among the 258 children in the control group (RD 0.0%, CI –2.0% to 2.0%; Supplementary Fig. 4). Two studies (268 children) focused on gastrointestinal adverse events and did not find significant difference between the groups (RD 2.0%, CI –1.0% to 5.0%; Supplementary Fig. 4). Evidence quality regarding the adverse events was ranked as low (Table 2).

## Discussion

In this systematic review and meta-analysis, we found low quality evidence that azithromycin does not reduce intensive care unit admissions. According to moderate quality evidence azithromycin reduces hospitalization time by –0.27 days. We also found that azithromycin does not prevent recurrent episodes of wheezing. Adverse events were similar between azithromycin and placebo.

Based on four studies azithromycin shortened the length of stay with –0.27 days—a difference that does not significantly benefit in the clinical practice. However, this was the only outcome in the review, where azithromycin showed efficacy and the evidence certainty was ranked as moderate. As the treatment durations in clinical practice have high variation, a mean reduction of six hours would not make an important impact, that would justify the addition of antibiotic to treatment of acute bronchiolitis. Need for intensive care unit admissions did not show evidence of a difference. However, the administration of azithromycin was started in hospital, and thus it is rather late. For the greatest theoretical anti-inflammatory and anti-viral benefit, azithromycin should be administered early at the onset of symptoms before the virus reaches its replication peak. Bacharier et al. included older children in their trial, where azithromycin was initiated immediately after symptom onset, it prevented the escalation of symptoms and reduced the need for hospitalization or corticosteroid treatment by 36%.^18^

Severe bronchiolitis episodes are associated with subsequent recurrent wheezing and asthma and passive immunization with recombinant anti-RSV antibody reduces this risk in late preterm infants.^25,26^ Azithromycin has been suggested to have the mechanistic rationale to benefit in the prevention of post-RSV recurrent wheezing too, because it inhibits neutrophilic airway inflammation, the dominant pattern seen in viral bronchiolitis.^16^ Azithromycin could also have an effect to aiway microbiota, and for example, prevent the harmful bacteria (such as Moraxella) associated and speculated to play an role in asthma development.^27–29^ On the other hand the use of antibiotics in early childhood, including during wheezing, is a known risk factor for asthma development.^30^ In this meta-analysis, four studies reported the recurrence of wheezing during their follow-ups from 3 months to 4 years, with no signal of a long-term protection with azithromycin.^9,18,21,30^ Although azithromycin has a long half-life because of its intracellular accumulation, maintaining measurable quantities in the airway macrophages for three weeks after the last dose of an 8-day course, recolonization of the airways seems inevitable after the treatment.^31^ Thus it seems unreasonable to expect any long-term protection against subsequent wheezing. This is in accordance with our finding that azithromycin could potentially decrease the recurrence of wheezing a few months onwards, but not up to 6 months or longer. A possible explanation for this could be that the short duration of given azithromycin treatment in the acute wheezing episode temporarily supresses local inflammation, but the effect vanishes. However, a repeated dosing or longer prophylactic courses would cause higher burden of antimicrobial resistance and influence both the microbiota in airways and gut also negatively.

Adverse events, which was the secondary outcome of this meta-analysis, were reported in 3 studies and they did not differ between the arms. Treatment related adverse event rates were approximately 6% in both the azithromycin and placebo groups. The detected gastrointestinal symptoms were mild diarrhea and vomiting, but the patients recovered and were able to continue the trial.

The prevalence of asthma increases with age. The immunology of asthma is heterogeneous; and the type of airway inflammation may be eosinophilic or neutrophilic.^32^ A recent Indian study found that elementary school aged children with poorly treated asthma might benefit from azithromycin along with standard treatment. They compared azithromycin (10 mg/kg) three times weekly for 3 months with standard treatment to standard treatment alone.^14^ Thus it could be possible that azithromycin would be beneficial in older children than included in this current meta-analysis.

The main weakness of this meta-analysis was the heterogeneity of the patients, ranging from mild wheezing to severe bronchiolitis requiring mechanical ventilation. Furthermore, due to heterogenous reporting, we were unable to estimate the impact of azithromycin based on the aetiology, and the lack of bacterial samples also is a limitation. RSV and non-RSV cases of wheezing are known to respond for example differently to oral corticosteroids. Further limitation is the low evidence quality as it varied between moderate and very low.

## Conclusion

Our meta-analysis, including seven double blinded RCTs, showed that azithromycin therapy on wheezing did not reduce subsequent wheezing episodes. The hospital length of stay was shorter compared to placebo. Adverse event rates were similar between the arms. Further studies with azithromycin administered already at onset of the respiratory infection symptoms are required to properly assess the antiviral and anti-inflammatory potential of makrolides with different dosings and viral etiology reporting is also warranted.

### Supplementary information

PRISMA 2020 Checklist

Supplementary Information

## Data Availability

All data generated during the review process are available upon request from the corresponding author.
