# Supplementary material for: Azithromycin for acute bronchiolitis and wheezing episodes in children – a systematic review with meta-analysis
Source: Pediatr Res. 2023 Dec 8;95(6):1441–7. doi: 10.1038/s41390-023-02953-z (PMC11126380; doi:10.1038/s41390-023-02953-z)
Supplement: Supplementary file 2 — Supplementary Information [file 41390_2023_2953_MOESM2_ESM.pdf]

Supplementary materials

Supplementary file 1

Search strategy in each database.

PubMed: ("azithromycin"[MeSH Terms] OR "azithromycin"[All Fields] OR "azithromycine"[All Fields] OR "azithromycin s"[All Fields]) AND ("bronchitis"[MeSH Terms] OR "bronchitis"[All Fields] OR "bronchitides"[All Fields] OR ("bronchiolitis"[MeSH Terms] OR "bronchiolitis"[All Fields] OR "bronchiolitides"[All Fields]) OR ("respiratory sounds"[MeSH Terms] OR ("respiratory"[All Fields] AND "sounds"[All Fields]) OR "respiratory sounds"[All Fields] OR "wheeze"[All Fields] OR "wheezes"[All Fields] OR "wheezing"[All Fields] OR "wheezed"[All Fields]) OR ("respiratory sounds"[MeSH Terms] OR ("respiratory"[All Fields] AND "sounds"[All Fields]) OR "respiratory sounds"[All Fields] OR "wheeze"[All Fields] OR "wheezes"[All Fields] OR "wheezing"[All Fields] OR "wheezed"[All Fields]))

Scopus: TITLE-ABS-KEY (azithromycin AND (bronchitis OR bronchiolitis)) AND (LIMIT-TO (LANGUAGE , "English")) AND (LIMIT-TO (DOCTYPE , "ar"))

Web Of Science: ALL=(azithromycin AND (bronchitis or bronchiolitis or wheeze or wheezing))

Supplementary figure 1 Need for hospital readmission during follow-up

Study or Subgroup	Azithromycin Events	Azithromycin Total	Control Events	Control Total	Weight	Risk Ratio M-H, Random, 95% CI
3.4.1 3 months follow-up						
Luisi et al 2020	3	37	3	33	4.8%	0.89 [0.19, 4.12]
McCallum et al 2013	9	50	8	47	15.0%	1.06 [0.45, 2.51]
**Subtotal (95% CI)**		**87**		**80**	**19.8%**	**1.01 [0.48, 2.15]**
Total events	12		11			
Heterogeneity: Tau² = 0.00; Chi² = 0.04, df = 1 (P = 0.85); I² = 0%						
Test for overall effect: Z = 0.04 (P = 0.97)						
3.4.2 6 months follow-up						
Luisi et al 2020	3	34	4	29	5.6%	0.64 [0.16, 2.63]
McCallum et al 2013	10	50	10	47	18.4%	0.94 [0.43, 2.05]
McCallum et al 2015	31	106	25	113	54.1%	1.32 [0.84, 2.08]
**Subtotal (95% CI)**		**190**		**189**	**78.1%**	**1.16 [0.79, 1.69]**
Total events	44		39			
Heterogeneity: Tau² = 0.00; Chi² = 1.28, df = 2 (P = 0.53); I² = 0%						
Test for overall effect: Z = 0.76 (P = 0.45)						
3.4.3 1 year follow-up						
Beigelman et al 2015	2	19	1	20	2.1%	2.11 [0.21, 21.36]
**Subtotal (95% CI)**		**19**		**20**	**2.1%**	**2.11 [0.21, 21.36]**
Total events	2		1			
Heterogeneity: Not applicable						
Test for overall effect: Z = 0.63 (P = 0.53)						
**Total (95% CI)**		**296**		**289**	**100.0%**	**1.14 [0.82, 1.60]**
Total events	58		51			
Heterogeneity: Tau² = 0.00; Chi² = 1.68, df = 5 (P = 0.89); I² = 0%						
Test for overall effect: Z = 0.78 (P = 0.44)						
Test for subgroup differences: Chi² = 0.37, df = 2 (P = 0.83), I² = 0%						

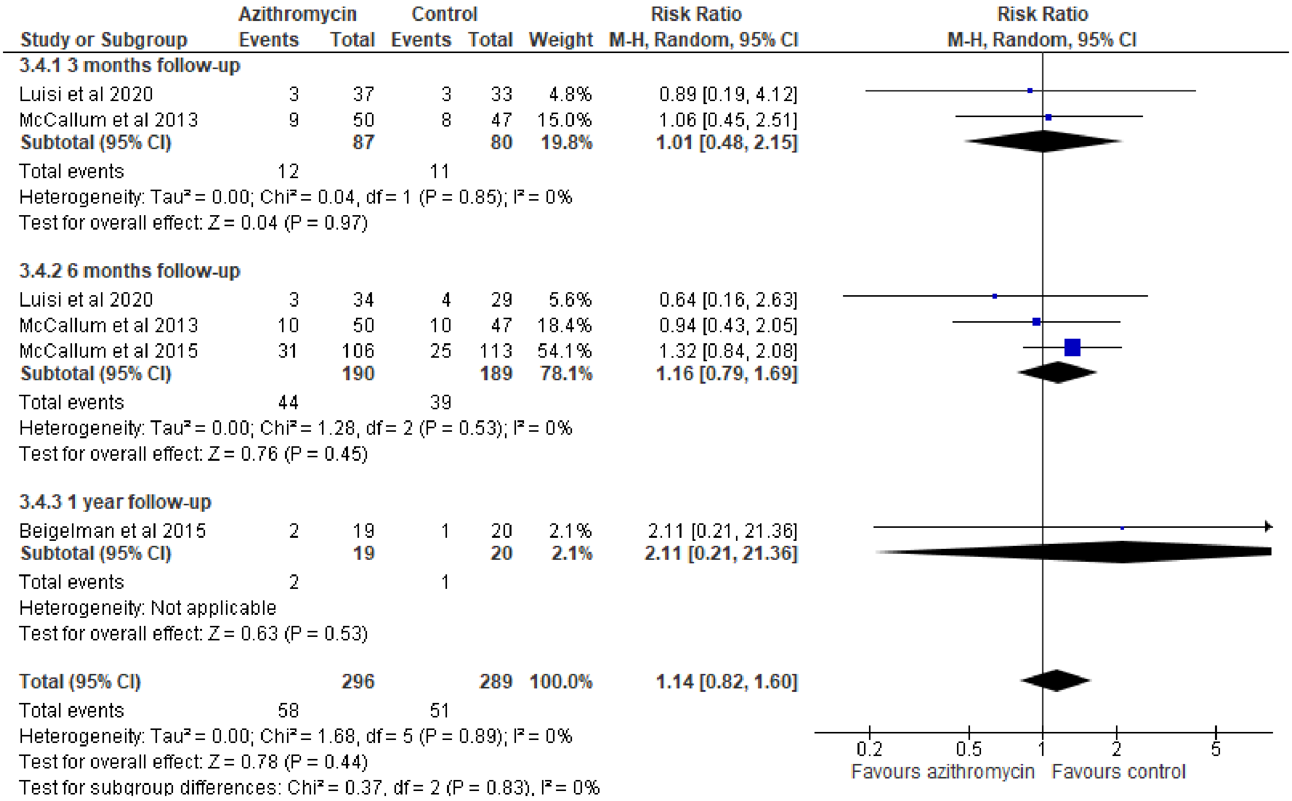

Supplementary figure 2 Wheeze recurrence (including those not needing hospital assessment) during follow-up (at least one episode)

Study or Subgroup	Azithromycin Events	Azithromycin Total	Control Events	Control Total	Weight	Risk Ratio M-H, Random, 95% CI
**1.5.1 3 months follow-up**						
Luisi et al 2020	7	37	13	33	27.6%	0.48 [0.22, 1.06]
**Subtotal (95% CI)**		**37**		**33**	**27.6%**	**0.48 [0.22, 1.06]**
Total events	7		13			
Heterogeneity: Not applicable						
Test for overall effect: Z = 1.82 (P = 0.07)						
**1.5.3 one year follow-up**						
Beigelman et al 2015	7	19	10	20	29.4%	0.74 [0.35, 1.54]
**Subtotal (95% CI)**		**19**		**20**	**29.4%**	**0.74 [0.35, 1.54]**
Total events	7		10			
Heterogeneity: Not applicable						
Test for overall effect: Z = 0.82 (P = 0.41)						
**1.5.4 2-4 years follow-up**						
Beigelman et al 2022	45	96	33	92	43.0%	1.31 [0.92, 1.85]
**Subtotal (95% CI)**		**96**		**92**	**43.0%**	**1.31 [0.92, 1.85]**
Total events	45		33			
Heterogeneity: Not applicable						
Test for overall effect: Z = 1.51 (P = 0.13)						
**Total (95% CI)**		**152**		**145**	**100.0%**	**0.84 [0.45, 1.56]**
Total events	59		56			
Heterogeneity: Tau² = 0.20; Chi² = 6.23, df = 2 (P = 0.04); I² = 68%						
Test for overall effect: Z = 0.56 (P = 0.58)						
Test for subgroup differences: Chi² = 6.19, df = 2 (P = 0.05), I² = 67.7%						

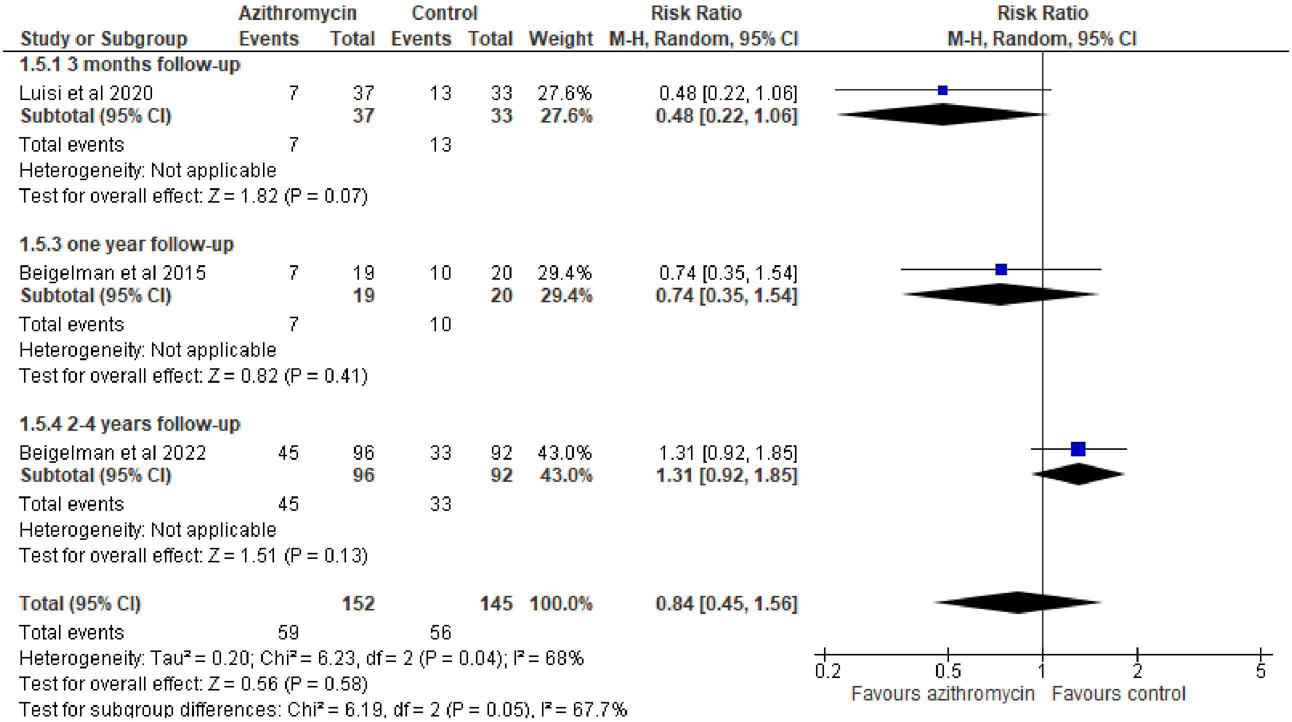

Supplementary figure 3 Asthma diagnosis during the follow-up

Study or Subgroup	Azithromycin Events	Azithromycin Total	Control Events	Control Total	Weight	Risk Ratio M-H, Random, 95% CI
**2.1.1 1 year follow-up**						
Beigelman et al 2015	2	19	5	20	36.4%	0.42 [0.09, 1.92]
**Subtotal (95% CI)**		**19**		**20**	**36.4%**	**0.42 [0.09, 1.92]**
Total events	2		5			
Heterogeneity: Not applicable						
Test for overall effect: Z = 1.12 (P = 0.26)						
**2.1.2 2-4 years follow-up**						
Beigelman et al 2022	14	96	9	92	63.6%	1.49 [0.68, 3.28]
**Subtotal (95% CI)**		**96**		**92**	**63.6%**	**1.49 [0.68, 3.28]**
Total events	14		9			
Heterogeneity: Not applicable						
Test for overall effect: Z = 0.99 (P = 0.32)						
**Total (95% CI)**		**115**		**112**	**100.0%**	**0.94 [0.29, 3.10]**
Total events	16		14			
Heterogeneity: Tau² = 0.42; Chi² = 2.11, df = 1 (P = 0.15); I² = 53%						
Test for overall effect: Z = 0.10 (P = 0.92)						
Test for subgroup differences: Chi² = 2.11, df = 1 (P = 0.15), I² = 52.5%						

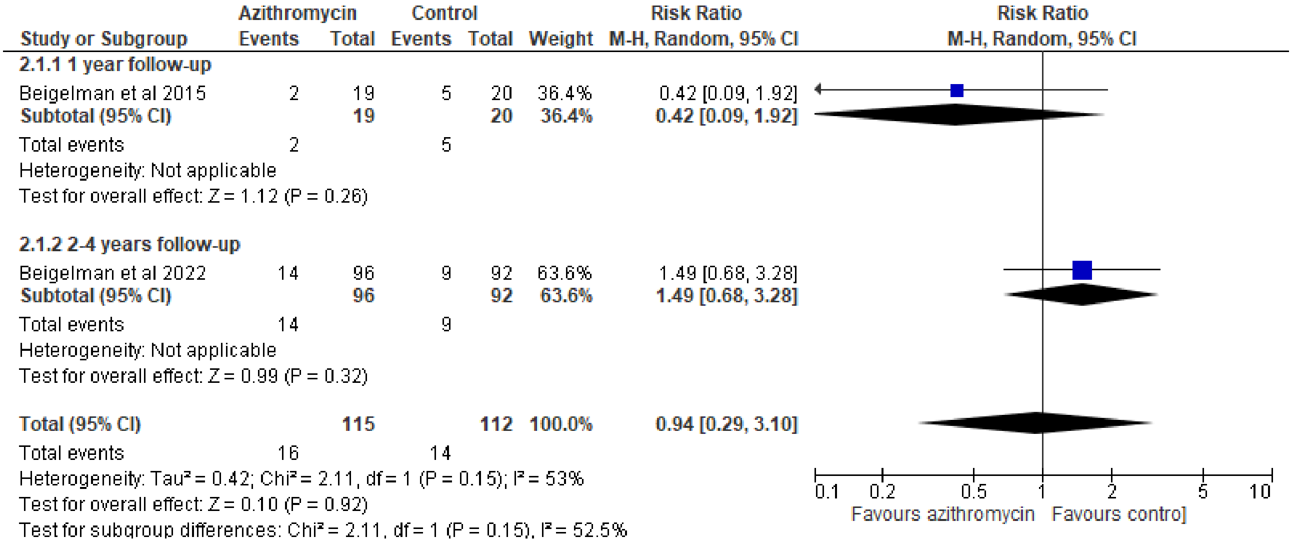

Supplementary figure 4 Adverse events

Study or Subgroup	Azithromycin Events	Azithromycin Total	Control Events	Control Total	Weight	Risk Difference M-H, Random, 95% CI
**4.1.1 Gastrointestinal adverse events**						
Beigelman et al 2015	7	19	8	20	1.0%	-0.03 [-0.34, 0.27]
McCallum et al 2015	2	106	0	113	99.0%	0.02 [-0.01, 0.05]
**Subtotal (95% CI)**		**125**		**133**	**100.0%**	**0.02 [-0.01, 0.05]**
Total events	9		8			
Heterogeneity: Tau² = 0.00; Chi² = 0.31, df = 1 (P = 0.58); I² = 0%						
Test for overall effect: Z = 1.16 (P = 0.24)						
**4.1.2 Treatment associated adverse events**						
Beigelman et al 2022	15	101	16	99	4.3%	-0.01 [-0.11, 0.09]
McCallum et al 2013	0	50	0	46	27.3%	0.00 [-0.04, 0.04]
McCallum et al 2015	1	106	1	113	68.3%	0.00 [-0.02, 0.03]
**Subtotal (95% CI)**		**257**		**258**	**100.0%**	**-0.00 [-0.02, 0.02]**
Total events	16		17			
Heterogeneity: Tau² = 0.00; Chi² = 0.26, df = 2 (P = 0.88); I² = 0%						
Test for overall effect: Z = 0.02 (P = 0.99)						

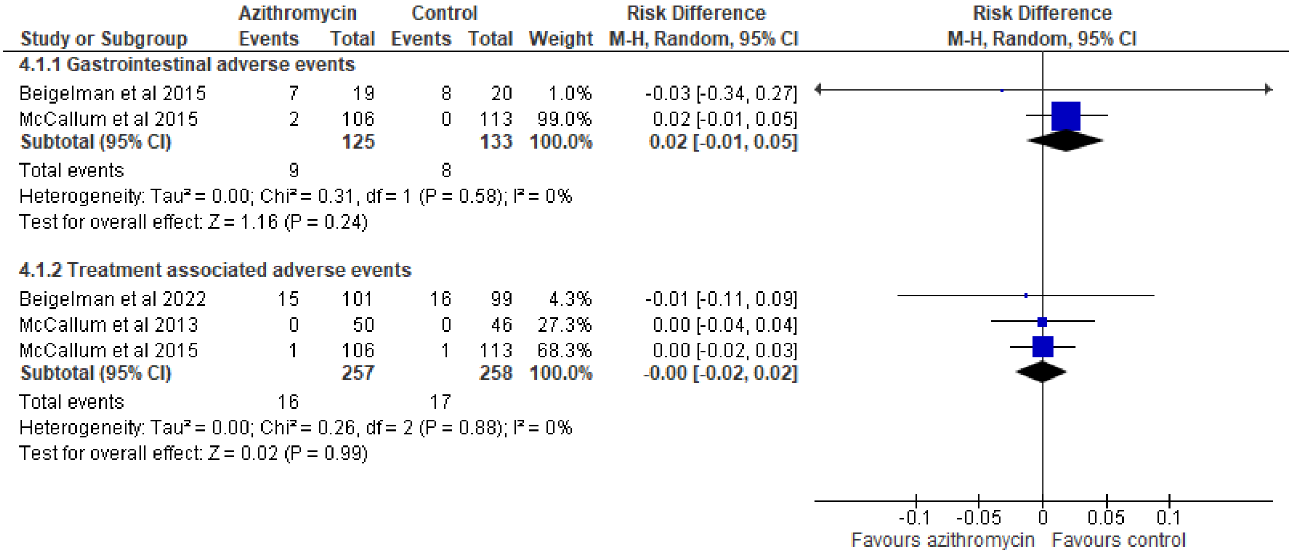

Supplementary table 1 Inclusion and exclusion criteria in the included studies.

Study	Inclusion criteria	Exclusion criteria
Kneyber et al 2007	Hospitalized infants with mild or moderate virologically confirmed clinical diagnosis of RSV<br>The clinical diagnosis of RSV was defined by a first attack of dyspnea and one or more symptoms compatible with lower respiratory tract infection including body temperature >37.58C, coughing, wheezing (audible with<br>or without stethoscope) and crackles on pulmonary auscultation	Symptoms restricted to upper respiratory tract infection<br>Apnea as the only symptom.<br>Nosocomial RSV<br>Patients were treated with antibiotics within 7 days prior to hospital admission.<br>Age > 24 months
Pinto et al	A diagnosis of bronchiolitis was confirmed if children were aged 12 months or younger and admitted to hospital with a clinical diagnosis of bronchiolitis (prodromal viral symptoms, with a first episode of wheezing/crackles, and tachypnea) and recruited within 48 hours of hospitalization and had a maximum of 72 hours of a history of lower respiratory tract	Any contraindication for oral macrolide therapy; prescription of macrolide therapy by the attending physician due to clinical and radiologic features consistent with a diagnosis of Chlamydia sp or *Bordetella pertussis* respiratory infection, having a chronic cardiopulmonary disorder, congenital or acquired immunodeficiency, neuromuscular disease, history of prematurity or other neonatal complications
McCallum et al 2013	≤18 months, admitted with a clinical diagnosis of bronchiolitis (according to standardised hospital protocols; ≤ 18 months, with cough and coryza, wheezing +/- crackles, respiratory distress with both tachypnoea (respiratory rate >50 beats/min) and retractions), required supplemental O2 and consented within 24 hrs of hospitalisation	Severe disease (admitted to intensive care unit); chronic lung disease, congenital heart disease, contraindications to macrolide use (e.g. liver dysfunction, hypersensitivity), diarrhoea (>2 stools of watery consistency more than normal pattern), received macrolides (in last 7-days), or clinical and radiological features consistent with a primary diagnosis of<br>pneumonia at time of randomisation
Beigelman et al 2015	1 to 18 months of age, otherwise healthy, hospitalized with a first episode of lower respiratory tract symptoms, nasopharyngeal swab result confirming infection with RSV, duration of respiratory symptoms from onset to admission of less than 5 days and randomization within 7 days of the onset of respiratory symptoms	History of previous wheeze, any<br>previous treatment with corticosteroid (systemic or inhaled), treatment with bronchodilators before the current RSV-induced bronchiolitis episode,<br>use of antigastroesophageal reflux medication, treatment with any antibiotics within the past 2 weeks (4 weeks for macrolide antibiotics),<br>prematurity (gestational age <36 weeks), or any chronic disease (lung,<br>cardiac, renal, or hepatic disease)
McCallum et al 2015	Aged ≤24 months and hospitalized with a standardized clinical diagnosis of bronchiolitis (age-adjusted tachypnea with wheeze or crackles), had parent-ascribed Indigenous ethnicity (Australian Aboriginal, Torres Strait Islander, Maori, and/or Pacific Islander), were consented within 24 h of hospitalization and had caregivers with a mobile phone	Severe disease (admitted to the intensive care unit); underlying chronic lung or congenital heart disease, contraindications to macrolides (e.g. hypersensitivity or liver dysfunction),diarrhoea (>2 two watery stools above the normal daily pattern), received macrolides within last seven-days, or clinical and radiographic features of a primary pneumonia. In New Zealand, infants<br>with previous wheezing illnesses were excluded due to the high incidence of asthma in Maori and Pacific Islander children.
Luisi et al 2020	Otherwise healthy infants hospitalized by acute bronchiolitis <12 months of age, recruited within 48 hours of hospitalization, with a maximum of 72 hours of a history of lower respiratory tract clinical signs (wheeze and/or respiratory distress).	Any restrictions to the use of oral macrolides, prescription of<br>macrolide therapy by the attending physician due to clinical and radiologic features consistent with a diagnosis of Chlamydia sp. or Bordetella pertussis<br>respiratory infection, a previous diagnosis of any chronic cardiopulmonary disorder, congenital/acquired immunodeficiency, or neuromuscular disease and a history of prematurity or other neonatal complications
Beigelman et al 2022	Otherwise healthy children, 1−18 months of age, who were admitted to St. Louis Children's Hospital with severe RSV bronchiolitis. RSV infection was confirmed by positive nasopharyngeal swab results by polymerase chain reaction and/or direct antigen detection. Severe bronchiolitis was defined by the presence of at least two of the following: respiratory rate >40 breaths per minute, cough, wheezing; the presence of rales, crackles, and/or rhonchi heard on chest auscultation; or paradoxical chest movements (retractions)	Prior wheeze, bronchiolitis, or asthma<br>diagnosis, significant medical history, prematurity, long duration of respiratory symptoms or mechanical ventilation, parents were not available, declined to participate
